# A Sensitivity-enhanced Fiber Grating Current Sensor Based on Giant Magnetostrictive Material for Large-Current Measurement

**DOI:** 10.3390/s19081755

**Published:** 2019-04-12

**Authors:** Shuchao Wang, Fu Wan, Hong Zhao, Weigen Chen, Weichao Zhang, Quan Zhou

**Affiliations:** 1 State Key Laboratory of Transmission & Distribution Equipment and Power System Safety and New Technology (Ministry of Education), School of Electrical Engineering, Chongqing University, Chongqing 400044, China; 15340582905@163.com (S.W.); fuwan@cqu.edu.cn (F.W.); weigench@cqu.edu.cn (W.C.); 2 State Key Laboratory of Engineering Dielectrics and Their Applications, School of Electrical and Electronic Engineering, Harbin University of Science and Technology, Harbin 150080, China; hongzhao@hrbust.edu.cn (H.Z.); weichao.zhang@hrbust.edu.cn (W.Z.)

**Keywords:** current sensor, fiber grating, finite element analysis, pressurization characteristics

## Abstract

Currently, in the modern power industry, it is still a great challenge to achieve high sensitivity and uninterrupted-online measurement of large current on the high voltage gridlines. At present, the fiber grating current sensors based on giant magnetostrictive material used in the modern power industry to achieve uninterrupted-online measurement of large currents on high voltage grid lines is a better method, but the sensitivity of this current sensor is relatively low, therefore, it is key to improve the sensitivity of this current sensor. Here we show a sensitivity-enhanced fiber grating current sensor based on giant magnetostrictive material (in the following, simply referred to as the sensitivity-enhanced fiber grating current sensor) that is able to achieve high sensitivity and uninterrupted-online measurement of large currents by means of pressurizing the giant magnetostrictive material. Sampling the power frequency sinusoidal alternating current signals with the amplitudes of 107, 157 and 262 A respectively, based on realistic factors, for the sensitivity-enhanced current sensor, the sensitivities, compared with that of the traditional fiber grating current sensor based on giant magnetostrictive material (in the following, simply referred to as the traditional fiber grating current sensor), were respectively enhanced by 268.96%, 135.72% and 71.57%. Thus the sensitivity-enhanced fiber grating current sensor allows us to solve the issue of high sensitivity and uninterrupted-online measurement of large currents that have been plaguing the power industry in a very simple and low-cost way.

## 1. Introduction

With the rapid development of the whole power industry, the highly automated and intelligent requirements of the current measurement system and the electrical appliances protection system are constantly increasing, therefore, the measurement of large currents has become an urgent problem to be solved [1,2,3,4,5,6,7,8,9,10,11,12,13]. For a long time, the current measurement of the high-voltage power grids has been done by using a traditional electromagnetic current sensor [11,12,13], but the traditional electromagnetic current sensor has various shortcomings, for example, magnetic saturation, ferromagnetic resonance, narrow frequency band, small dynamic range, oil flammability and explosivity, etc. [11]. Therefore, this electromagnetic current sensor has been far from meeting the needs for online detection, high-precision fault diagnosis, and power digital networks of the new generation power systems, and in this case, it is imperative to seek a new type of this current sensor [5,6,7,8,9,10,11,12]. After many years of development, optical fiber current sensor technology is applied in the power industry to achieve uninterrupted-online measurement of large current on the high voltage grid lines has gradually become the mainstream of the current power industry [9,10,11,12,13]. The optical fiber current sensor has many advantages, for example, this current sensor doesn’t produce magnetic saturation and ferromagnetic resonance, and at the same time, it has a large dynamic range, which enables this current sensor to produce a high linearity response over a large dynamic range, besides, the sensor also has the advantages of anti-electromagnetic interference, good insulation, small size, lightweight and so on, more importantly, there is no danger of high voltage caused by secondary open circuit and oil leakage and explosions of this traditional oil-filled current sensor [11,12].

At present, the mainstream optical fiber current sensors for uninterrupted-online measurement of large currents on high voltage gridlines mainly include three types, namely, the photoelectric hybrid current sensor, the current sensor based on Faraday magneto-optical effect and the current sensor based on giant magnetostrictive material [5,6,7,8,9,10,11,12,13]. Among these three types, the optical fiber current sensor based on giant magnetostrictive material shows more advantages than another two types of current sensors. For the photoelectric hybrid current sensor, there is no better solution to the power supply problem of high-potential electronic circuit and the reliability problem of electronic circuit [11]. Meanwhile, for the current sensor based on Faraday magneto-optical effect, the problem of output waveform distortion caused by birefringence hasn’t been solved very well [11,12]. The sensing core of the optical fiber current sensor based on giant magnetostrictive material is the fiber Bragg grating (FBG) and the giant magnetostrictive material (GMM) [11,12,13]. After many years of research and development, FBG sensing technology has become the most reliable and practical sensing technology in optical fiber sensing technology [9,10,11,12]. FBGs can only sense strain and temperature, and are completely insensitive to other physical quantities, therefore, due to the above advantages, FBG sensing technology has become the preferred method for uninterrupted-onlinemeasurement of large current on the high voltage gridlines [11]. The fiber grating current sensor based on giant magnetostrictive material is the application of FBG sensing technology, thus it is a better method that the fiber grating current sensor based on giant magnetostrictive material is used in the modern power industry to achieve uninterrupted-online measurement of large current on the high voltage grid lines, but the sensitivity of this current sensor is relatively low, therefore, it is key to improve the sensitivity of this current sensor [10,11,12,13].

In the past decade, the research in the field of improving the sensitivity of the fiber grating current sensor has made some progress [1,2,3,4,5,6,7,8,9,10,11,12]. Generally speaking, there are two ways to improve the sensitivity: one is to improve the demodulation system of the fiber grating current sensor. The other is to improve the main body of the fiber grating current sensor [11,12]. In the improvement of the demodulation system, the most widely used way is to improve the demodulation method, the demodulation methods of FBG include the sideband demodulation method, interference demodulation method and matched demodulation method, etc. [5,6,7,8,9,10,11,12]. Among them, the interference demodulation method has higher sensitivity [11], but it has a very complicated structure and is susceptible to the external environment, and at the same time, the stability of the system is poor [10,11,12,13]. For the sideband demodulation method [11], the most commonly used demodulation system composed of the tuned Fabry-Perot etalon is very complicated, and the repeatability of the wavelength and driving voltage corresponding to the adjustable Fabry-Perot cavity is poor, only for static measurement [3,7,8,9,10]. The matched demodulation method has also high sensitivity [7,8,9,10,11,12], but it has a narrow dynamic range and is susceptible to the external environment, at the same time, the cost of CWDM and AWG is very high as well [11]. In the research of this sensor body [11,12], universities, research institutes and many enterprises at home and abroad are devoted to the development of giant magnetostrictive materials with better flexibility [1,2,3,4,5,6,7,8,9,10,11,12,13], the use of more accurate reading and conversion instruments, the selection of more accurate and suitable error processing methods and writing more optimized the structural data processing algorithms, etc. [11,12]. However, there, up to now, are various problems and defects in these methods. So far, there is no way from the structural point of the sensor probe in the cheap and simple way to solve the problem of high sensitivity measurements of large currents, which has been plaguing the power industry.

## 2. Materials and Methods

In this paper, we propose a new type of the sensor-probe structure which enhances the sensitivity of the fiber grating current sensor by means of pressure enhancement. It is well known that this fiber grating current sensor is based on FBG and GMM [11,12,13].

**Figure 1:**
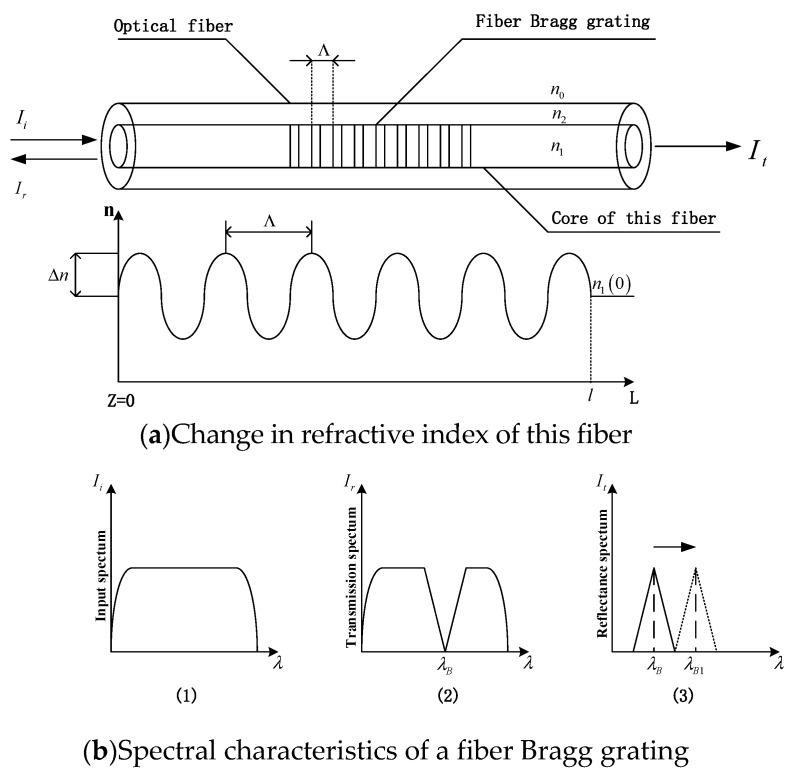
The structure and spectral characteristics of FBG. (**a**) *I_i_* is the incident light intensity that is incident into FBG. *I_r_* is the reflected light intensity reflected by FBG. *I_t_* is the transmitted light intensity through FBG. Λ is the grating period. (**b**) (1) represents the incident spectrum of the incident into FBG. (2) represents the transmission spectrum through FBG. (3) represents the reflected spectrum reflected by FBG. *λ_B_* is the wavelength of the reflected wave. *λ_B_*_1_ represents the wavelength of the reflected wave after the fiber grating is externally affected.

A FBG is composed of filters and mirrors that exist inside the fiber core, as shown in Figure 1a [11]. Since the refractive index of the fiber grating itself is periodically changed, then when a laser beam is incident from one side of FBG, and optical coupling occurs during forward and backward transmission, when the Bragg equation *λ_B_* = 2*n_eff_*Λ is satisfied, the laser transmitted backward is coupled by the laser of wavelength *λ_B_*, thus it finally forms a new laser, and the peak of the wavelength of the laser is *λ_B_*. Most of the laser beam can form the transmission spectrum, as shown in Figure 1b [11].

When the external physical quantity acts on the FBG, the *n_eff_* and Λ of the fiber core can change correspondingly, which can cause the central wavelength of the FBG to shift, which is why FBG can be used as a sensing material.

In addition, there are two basic indicators for the characterization of GMM as well. One is the magnetostriction coefficient *γ*. It indicates that the magnetostriction coefficient describes the extent to which the entire magnet changes in unit length along the magnetization direction during magnetization.

The other is the dynamic magnetostriction coefficient *d*_33_. It describes the sensitivity of giant magnetostrictive materials to magnetic field, this relationship is specifically expressed as:

(1) $$d_{33}=\frac{d\gamma }{dH}$$

In the formula, *γ* is the magnetostriction coefficient of this material, *H* is the strength of the magnetic field in which the material is located [11,12].

**Figure 2:**
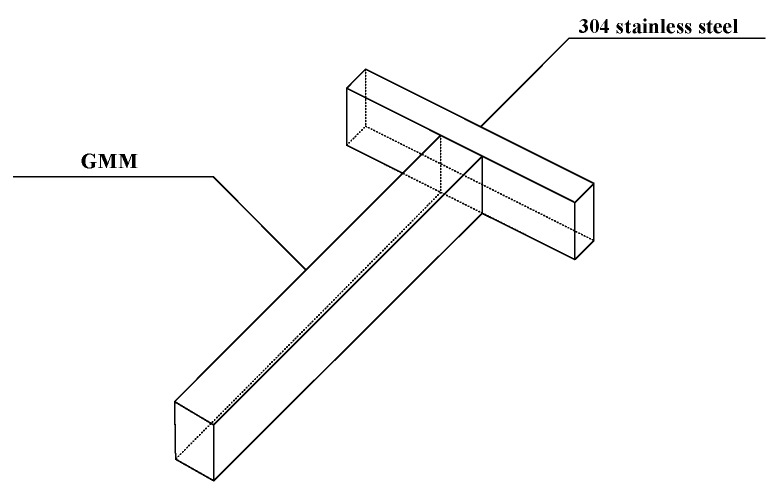
The T-shape structure composed of GMM and 304 stainless steel. The bracket of the T-shape structure is GMM, which is called Terfenol-D rod. The beam of the T-shape structure is 304 stainless steel.

Based on a large number of previous studies on this material [1,2,3,4,5,6,7,8,9,10,11,12,13,14,15,16,17,18,19,20,21,22,23,24,25,26,27,28], the sensitivity of GMM, which is called Terfenol-D rod [25,26,27,28], to magnetic field is related to the prestressing force applied to GMM [14,15,16,17,18,19,20,21,22,23,24,25,26,27,28]. In the case different sizes of prestressing force are applied to this material, the slope of the magnetostriction coefficient of GMM, compared with the case of no prestressing force at all, is significantly improved, namely, the dynamic magnetostriction coefficient of GMM is significantly improved [14,15,16,17,18,19,20,21,22,23,24,28]. That is to say, in the case prestressing force is applied, the sensitivity of GMM to the magnetic field, compared to the absence of prestressing force, is significantly enhanced. Therefore, we took advantage of this property of GMM to enhance the sensitivity of large current measurement on the high voltage gridlines [14,15,16,17,18,19,20,21,22,23,24,25,26,27,28]. A beam was added to the conventional cuboid GMM to withstand the applied pressure, thus a T-shape structure was formed. Because the beam does not effect the change of the magnetic field, the material of this beam has to be a nonmagnetic material, at the same time, this material should be wearable, corrosion resistant, not easy to rust, common and cheap as well. Therefore, based on the above requirements, the most common 304 stainless steel on the market was selected as the material for this beam [29]. The specific design is shown in Figure 2.

The T-shape structure was placed in a copper support structure. A special copper screw was placed on the upper right side of the structure to pressurize the beam of the T-shape structure by screwing, so as to achieve the goal of pressurizing the T-shape structure.

**Figure 3:**
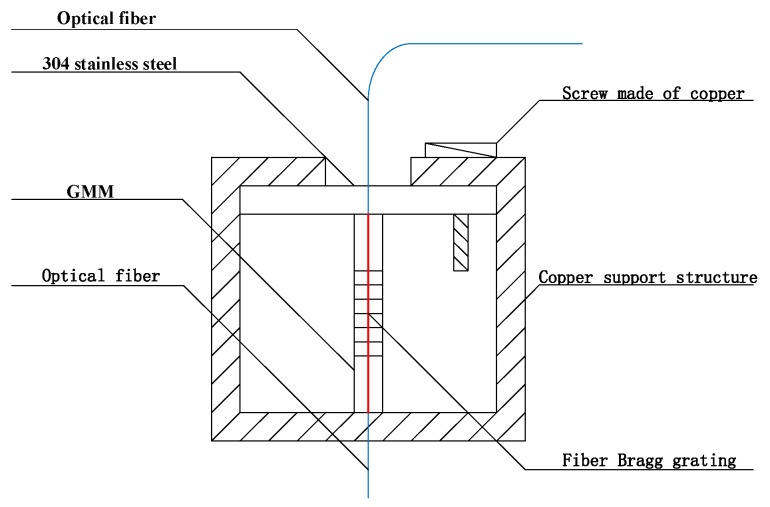
The sensor probe of this sensitivity-enhanced fibergrating current sensor. The central wavelength of the FBG is about 1550 nm.

**Figure 4:**
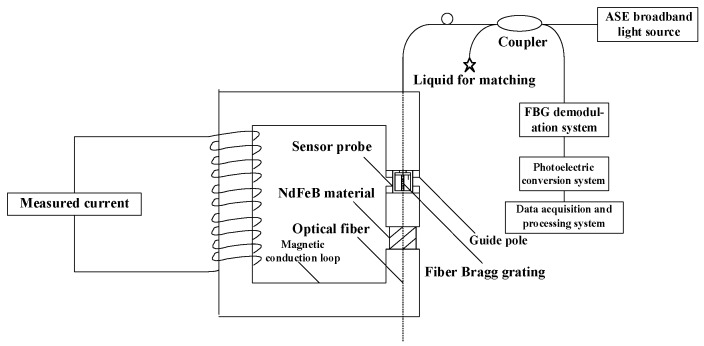
The overall structure of the sensitivity-enhanced fiber grating current sensor. The magnetic conduction loop is constructed of ferrite material. The sensing system is composed of a magnetic circuit system established by a magnetically conductive material and a guide pole. The NdFeB material provides the required bias magnetic field. An ASE broadband light source is used as the incident source in this sensor. The coupling ratio of the coupler is 50:50.

Therefore, the sensor probe of the sensitivity-enhanced fiber grating current sensor was constituted with GMM, FBG, the copper support structure and the screw made of copper together. The specific structure is shown in Figure 3. In this sensor probe, the FBG was normally attached to the centerline of GMM. The way we paste FBG on GMM on which a V-shaped groove was cut, firstly, along the axial direction of one side of GMM, which is called Terfenol-D rod, then the FBG was placed in the groove and pre-tensioned, finally, the FBG was pasted onto GMM. Therefore, it can be considered here that GMM is integrated with the FBG [9,10,11,12,13,14,15,16,17,18,19,20,21,22,23,24,25,26,27,28]. Then, after applying pressure to the GMM, the GMM sensitivity to the magnetic field was significantly improved, namely, it can be considered that the sensitivity of the sensor probe had been significantly improved, which enhances the sensitivity of the overall fiber grating current sensor. The overall structure of the sensitivity-enhanced fiber grating current sensor is shown in Figure 4. The sensor itself is composed of a laser source system, a sensing system, a FBG demodulation system, a photoelectric conversion system and a data acquisition and processing system.

In general, when the measured current signal is sensed by the FBG, the relationship between the measured power frequency sinusoidal alternating current signal and the reflected central wavelength of the FBG, namely, the optical signal, can reflect the results of this experiment, however, in order to get clearer and easier to understand results, in this experiment, the optical signal was converted by the photoelectric conversion system into a sinusoidal alternating current signal, which saved the characteristics of the measured power frequency sinusoidal alternating current signal, however, the values became smaller than the measured current signal, to reflect the results of this experiment. Therefore, the working principle of the sensitivity-enhanced fiber grating current sensor is that the measured power frequency sinusoidal alternating current signal generates an excitation magnetic field which causes the continuous change of the magnetic field. In turn, the GMM will also continue to expand with the change of the magnetic field. The center wavelength of FBG will change continuously as well. Then, the information of the FBG center wavelength change is demodulated by the demodulation system, and then the demodulated optical signal passes through the photoelectric conversion system, thereby converting the optical signal into the current signal, finally, the current signal is passed into the data acquisition and processing system so that the feature of the measured power frequency sinusoidal alternating current signal is restored. Finally, through this way, the purpose of measuring large current can be achieved.

**Figure 5:**
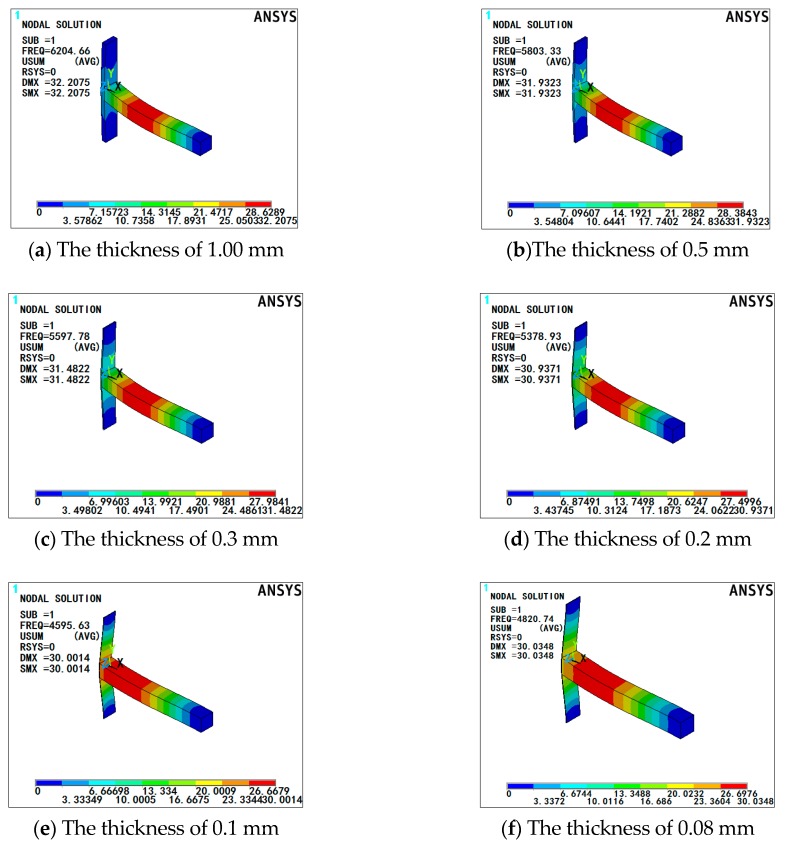
The specific simulation results of the T-shape structure composed of 304 stainless steel of different thicknesses and GMM. (**a**) The thickness of 304 stainless steel is 1.00 mm. (**b**) The thickness of 304 stainless steel is 0.50 mm. (**c**) The thickness of 304 stainless steel is 0.30 mm. (**d**) The thickness of 304 stainless steel is 0.20 mm. (**e**) The thickness of 304 stainless steel is 0.10 mm. (**f**) The thickness of 304 stainless steel is 0.08 mm.

In addition, for any type of sensor, the sensor probe is the most important core component. Therefore, the size of the sensor probe of this sensitivity-enhanced fiber grating current sensor is especially important. Initially, it needs to be clear that the size of this whole sensor probe of this sensitivity-enhanced fiber grating current sensor cannot be too large, because the sensor probe needs to collect the magnetic field. Due to the limitation of the size of the magnetic conduction loop and the copper support structure [10,11,12,13,28], normally the GMM size was selected as 25.00 mm × 3.00 mm × 3.00 mm. In order to match GMM, the width of 304 stainless steel part was also selected as 3.00 mm. Meanwhile, due to the limitation of the width of the guide pole, the magnetic conduction loop and the copper support structure [11,12,13], the length of 304 stainless steel part was selected as 22.00 mm. Besides, it is most important to choose the thickness of 304 stainless steel. Similarly, due to the limitation of the size of the magnetic conduction loop [10,11,12,13,28], normally, the thickness of 304 stainless steel cannot exceed 1.00 mm. At the same time, if the thickness of this 304 stainless steel piece is too large, it is highly probable that there will be a situation where this 304 stainless steel hinders the movement of the GMM, which causes the sensitivity of the fiber grating current sensor not to increase, but decrease. Therefore, in order to avoid this happening, under the condition that other factors remain the same, T-shape structures composed of 304 stainless steel of different thickness and GMM were simulated by the ANSYS simulation software, so as to select a thickness dimension of 304 stainless steel to reduce the extent of the 304 stainless steel obstruction of GMM movement as much as possible. The specific simulation results of the T-shape structure by the ANSYS simulation software are shown in Figure 5.

It can be seen from the simulation results in Figure 5 that 304 stainless steel with a thickness of 0.1 mm and 304 stainless steel with a thickness of 0.08 mm were more curved, namely, 304 stainless steels of 0.10 mm and 0.08 mm thickness were less obstructive to GMM movement. Due to some practical factors such as the stability of the sensor probe, the difficulty of machining, durability and so on [21,22,23,24], the thickness of 304 stainless steel was selected as 0.10 mm. Consequently, the size of 304 stainless steel was 22.00 mm × 3.00 mm × 0.10 mm.

**Figure 6:**
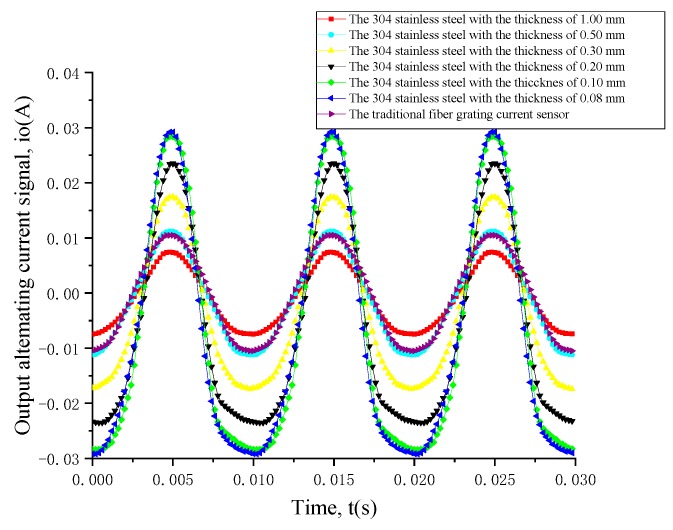
The experimental results of this sensitivity-enhanced fiber grating current sensor with different 304 stainless steel thicknesses and this traditional fiber grating current sensor together between output alternating current signal (*i_o_*) and time (*t*). The red curve, the light blue curve, the yellow curve, the black curve, the green curve and the blue curve show the relationship of the sensitivity-enhanced fiber grating current sensor, whose 304 stainless steel thicknesses are, respectively, 1.00, 0.50, 0.30, 0.20, 0.10 and 0.08 mm. The purple curve shows the relationship of the traditional fiber grating current sensor, in which the 304 stainless steel doesn’t exist, whose GMM in the sensor probe is the same as that of the sensitivity-enhanced fiber grating current sensor in size or other aspects.

To better prove the result obtained by the above simulation, we implemented an experiment to prove the correctness of this simulation. Under the condition that other factors remain the same apart from the thicknesses of 304 stainless steel in this simulation. According to the references related to GMM [11,12,13,14,15,16,17,18,19,20,21,22,23,24,25,26,27,28], a force of 5 MPa was selected to apply to the GMM. In order to simulate the force process in this simulation, the power frequency sinusoidal alternating current signals with the amplitude of 100 A were applied as the excitation source to implement the experiment for proving the correctness of this simulation. Thus the experimental results of the output alternating current signals of these sensitivity-enhanced fiber grating current sensors with 304 stainless steel of different thicknesses and this output alternating current signal of the traditional fiber grating current sensor, in which GMM in the sensor probe was the same as that of the sensitivity-enhanced fiber grating current sensor are obtained, and were compared to prove the correctness of this simulation. The specific experimental results of the comparison of the seven sensitivities are shown in Figure 6.

It can be seen from Figure 6 that in this situation that the sensitivity of the sensitivity-enhanced fiber grating current sensor, whose GMM was pressured, is lower than that of this traditional fiber grating current sensor has really occurred when the thickness of 304 stainless steel is 1.00 mm. When the thickness of 304 stainless steel is 0.50 mm, the sensitivity of this sensitivity-enhanced fiber grating current sensor is basically the same as the sensitivity of this traditional fiber grating current sensor. Therefore, the previous concerns seem to be very necessary, this situation that the 304 stainless steel hindered the expansion of GMM had indeed occurred. It can be also seen from Figure 6 that the sensitivity from the sensitivity-enhanced fiber grating current sensor where the thickness of 304 stainless steel is 1.00 mm to the sensitivity-enhanced fiber grating current sensor where the thickness of 304 stainless steel is 0.10 mm is gradually increasing, therefore, this has also proved the importance of choosing this thickness of 304 stainless steel. Besides, it can be also seen from Figure 6 that the sensitivity of the sensitivity-enhanced fiber grating current sensor where the thickness of 304 stainless steel is 0.10 mm is basically the same as that of the sensitivity-enhanced fiber grating current sensor where the thickness of 304 stainless steel is 0.08 mm as well, and one difference is that the amplitude of output alternating current signal of the sensitivity-enhanced fiber grating current sensor where the thickness of 304 stainless steel is 0.08 mm is a little larger than that of the sensitivity of the sensitivity-enhanced fiber grating current sensor where the thickness of 304 stainless steel is 0.10 mm. This also obeyed the rule that the smaller thickness of 304 stainless steel of this sensitivity-enhanced fiber grating current sensor, the smaller the hindrance to the movement of GMM. At the same time, the various realistic factors were considered, for example, the stability of this sensor probe, the difficulty of machining, durability and so on. This was in line with the actual situation that the size of 304 stainless steel was selected as 22.00 mm × 3.00 mm × 0.10 mm.

Besides, in order to be able to perform quantitative analysis, the size of the prestressing force on the sensor probe had to be calibrated. The method for calibrating the prestressing force in this experiment was that the GMM which was the same as the GMM in T-shape structure was applied with a certain amount of pressure. Then an optical fiber F-P current sensor was placed on the surface of this GMM, at the same time, the optical fiber F-P current sensor was connected to a fiber strain gauge, and this fiber strain gauge was connected to a computer with dedicated software. After applying a certain amount of pressure, the cavity of this optical fiber F-P current sensor adhered to this GMM changed, and the changed length of this cavity can be obtained and be recorded by this fiber stain gauge and this computer with dedicated software. Similarly an optical fiber F-P current sensor, which was exactly the same as this optical fiber F-P current sensor adhered to this above GMM, was placed on the surface of this GMM which existed in the T-shape structure sensor probe as well, and the optical fiber F-P current sensor was also connected to a fiber strain gauge, then this fiber strain gauge was connected to a computer with dedicated software as well. This GMM in T-shape structure was applied the prestressing force by means of screwing, then the changes of the cavity length were recorded on this computer, when the changed length of this cavity matches the recorded length of the cavity which was obtained by applying the pressure only to this GMM, the pressures applied on the two GMM were the same. Then the pressure calibration was completed by engraving the marks on this screw [10]. Due to only a proof experiment and some actual complicated factors, the size of the prestressing force was only calibrated when the pressure is 1 MPa.

**Figure 7:**
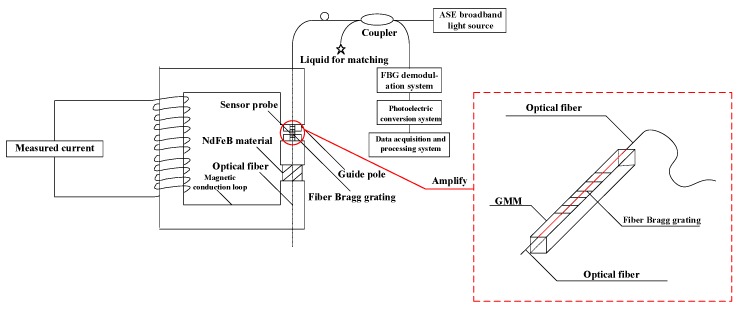
The overall structures of the traditional fiber grating current sensor and the senor probe composed of only FBG and GMM. The magnetic conduction loop is constructed of ferrite material and the sensor probe. The sensing system is composed of a magnetic circuit system established by a magnetically conductive material and a guide pole. The NdFeB material provides the required bias magnetic field. The ASE broadband light source is used as the incident source in this sensor. The coupling ratio of the coupler is 50:50.

In this article, the sensitivity-enhanced fiber grating current sensor has been comparing with the traditional fiber grating current sensor. Virtually, the overall structure of the traditional fiber grating current sensor is basically the same as that of the sensitivity-enhanced fiber grating current sensor [11,12,13,28]. The biggest difference between the two sensors is the different structure of the sensor probe. The sensor probe structure of the traditional fiber grating sensor is composed of GMM with a rectangular parallelepiped structure and FBG, and FBG is similarly attached to the centerline of GMM, which is called Terfonol-D rod [9,10,11,12]. In the traditional sensor probe structure, GMM is free from any prestressing force [8,9,10,11,12,13,28]. The overall structure of the traditional fiber grating current sensor and the senor probe structure composed of only FBG and GMM are showed in Figure 7.

## 3. Results

For power transmission lines with the rated voltage of 35kV and the transmission capacity of 6500kW, which is usually used as the power transmission lines between provinces and provinces, the maximum transmission current is 107.22 A [30,31]. For transmission lines with the rated voltage of 110kV and the transmission capacity of 30000kW, which is usually used as the power transmission lines between cities and cities, the maximum transmission current is 157.46 A [30,31]. For transmission lines with the rated voltage of 220 kV and the transmission capacity of 100,000 kW, which is usually used as the power transmission lines between communities and communities, the maximum transmission current is 262.43 A [30,31]. Therefore, simulating the above transmission lines in a laboratory environment, namely, the sensitivity-enhanced fiber grating current sensor and the traditional fiber grating current sensor were simultaneously used to measure power frequency sinusoidal alternating current signals with the amplitude of 107, 157and 262 A. It needs to be clearly emphasized that this sensitivity-enhanced fiber grating current sensor and this traditional fiber grating current sensor were placed in the same laboratory where many air conditioners were controlled to keep this laboratory at a constant temperature for this experimentation, and in this study, the sensor probes of the two sensors were equipped with a temperature test system, the purpose of which was to keep the temperature around the two sensor probes the same as possible, which ensured that this sensitivity-enhanced fiber grating current sensor and this traditional fiber grating current sensor were affected by the same temperature. Therefore, in this way, the experimental results were only related to the pressures, which made the results more universal [1,10,11,12,13,28].

**Figure 8:**
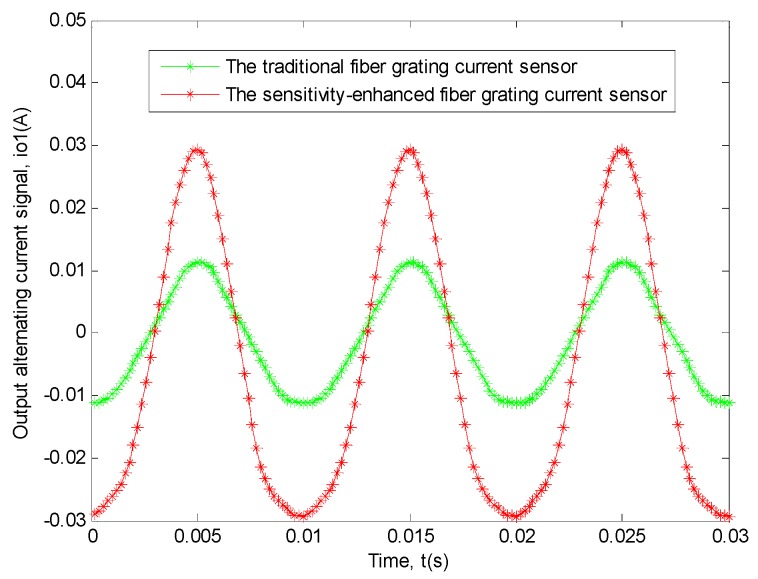
This experiment result of the two sensors between output alternating current signal (*i_o_*_1_) and time (*t*). The red curve shows the relationship of the sensitivity-enhanced fiber grating current sensor. The green curve shows the relationship of the traditional fiber grating current sensor.

For the measurement of the power frequency sinusoidal alternating current signal with the amplitude of 107 A, we had done ten experiments and calculated the average of the data from the ten experiments. And the relationship based on the actual average data obtained by the two sensors between output alternating current signal and time is shown in Figure 8.

**Figure 9:**
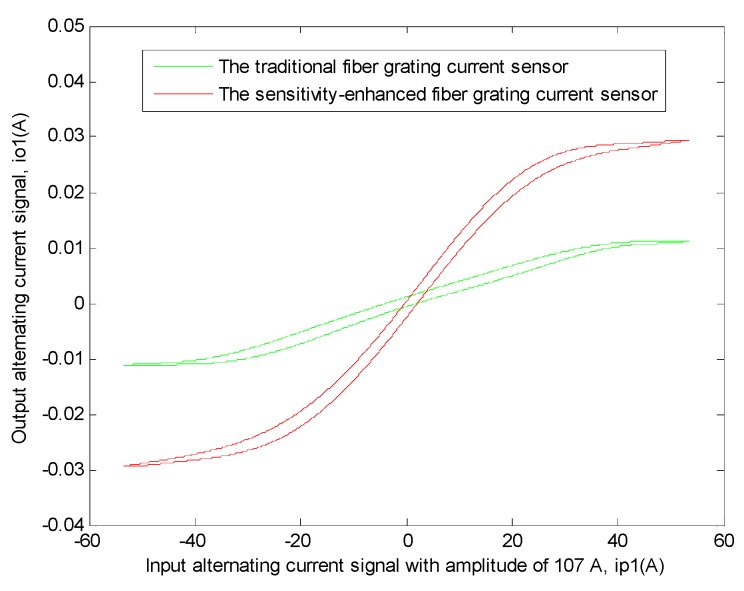
The transfer function relationship curves of the two sensors between output alternating current signal (*i_o_*_1_) and input alternating current signal with amplitude of 107 A (*ip_o_*_1_), respectively. The red curve shows the transfer function relationship of the sensitivity-enhanced fiber grating current sensor. The green curve shows the transfer function relationship of the traditional fiber grating current sensor.

In order to explain this problem better, the transfer function relationship curves of the two sensors between the input signal and the output signal need to be drawn [11,12,32,33,34,35,36,37,38]. We all know that the measured current signal was the power frequency alternating sinusoidal current signals, and it can be clearly seen from Figure 8 that the output current signal was also similar to the power frequency alternating sinusoidal current signal, thus the relational expression between the input current signal and time and the relational expression between the output current signal and time can be directly fitted by MATLAB, then it can be known from the obtained results and the output current signal and the input current signal are directly related to time, therefore, the relationship between the input current signal and the output current signal, which is similar to Lissajous Figure, can be directly drawn by MATLAB, besides, in order to compare only the slopes of the linear regions of the two graphs, the two graphs are moved to the origin place of the coordinate system [11,12,28]. The specific situation is shown in Figure 9.

Then, the data corresponding to different periodic linear regions in the experimental result of two sensors were extracted and their average values were calculated [10,11,12,13,28]. The linear region relationship equations of the two sensors between this input current signal and this output current signal can be obtained by linear fitting with this related software. Their equations are as follows.

This sensitivity-enhanced fiber grating current sensor:

(2) $$Y_{1}\left(x_{1}\right)=0.001108x_{1}$$

Here, when these data are linearly fitted, the value of *R*^2^ is 0.9987, which indicates the best level of fitting at this moment.

This traditional fiber grating current sensor:

(3) $$Y_{2}\left(x_{2}\right)=0.0003003x_{2}$$

Here, when these data are linearly fitted, the value of *R*^2^ is 0.9975, which indicates the best level of fitting at this moment.

It can be seen from the above two equations that the linear region slope of the transfer function of the sensitivity-enhanced fiber grating current sensor is 0.001108, and at the same time, the linear region slope of the transfer function of the traditional fiber grating current sensor is 0.0003003. Then the sensitivity of the sensitivity-enhanced fiber grating current sensor is 268.96% higher than that of the traditional fiber grating current sensor.

**Figure 10:**
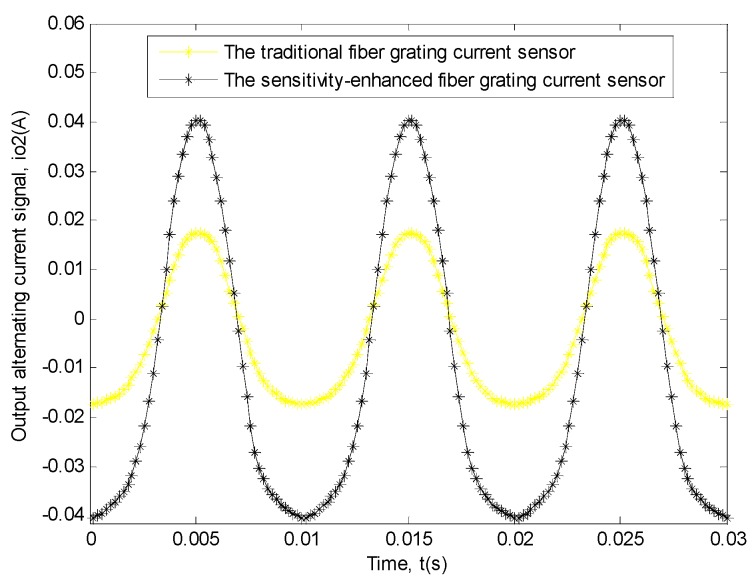
This experiment result of the two sensors between output alternating current signal (*i_o_*_2_) and time (*t*). The black curve shows the relationship of the sensitivity-enhanced fiber grating current sensor. The yellow curve shows the relationship of the traditional fiber grating current sensor.

For the measurement of the power frequency sinusoidal alternating current signal with the amplitude of 157 A, we had done ten experiments and calculated the average of the data from the ten experiments as well. And the relationship based on the actual average data obtained by the two sensors between output alternating current signal and time is shown in Figure 10.

**Figure 11:**
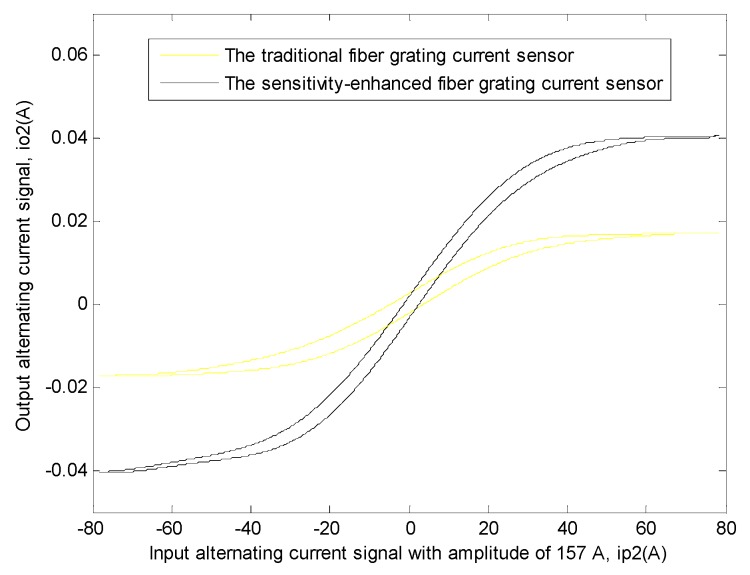
The transfer function relationship curves of the two sensors between output alternating current signal (*i_o_*_2_) and input alternating current signal with amplitude of 157 A(*i_p_*_2_), respectively. The black curve shows the transfer function relationship of the sensitivity-enhanced fiber grating current sensor. The yellow curve shows the transfer function relationship of the traditional fiber grating current sensor.

Similarly, in order to explain this problem better, the transfer function relationship curves of the two sensors between this input signal and this output signal need to be drawn [11,12,32,33,34,35,36,37,38], and the method was the same as that used in the first experiment. The specific situation is shown in Figure 11.

Then, the data corresponding to different periodic linear regions in the experimental result of two sensors were also extracted and their average values were calculated [10,11,12,13,28]. The linear region relationship equations of the two sensors between input current signal and output current signal can be obtained by linear fitting with this related software. Their equations are as follows.

This sensitivity-enhanced fiber grating current sensor:

(4) $$f_{1}\left(x_{1}\right)=0.001234x_{1}$$

Here, when these data are linearly fitted, the value of *R*^2^ is 0.9987, which indicates the best level of fitting at this moment.

This traditional fiber grating current sensor:

(5) $$f_{2}\left(x_{2}\right)=0.0005235x_{2}$$

Here, when these data are linearly fitted, the value of *R*^2^ is 0.9988, which indicates the best level of fitting at this moment.

It can be seen from the above two equations that the linear region slope of the transfer function of the sensitivity-enhanced fiber grating current sensor is 0.001234, and at the same time, the linear region slope of the transfer function of the traditional fiber grating current sensor is 0.0005235. Then the sensitivity of the sensitivity-enhanced fiber grating current sensor is 135.72% higher than that of the traditional fiber grating current sensor.

**Figure 12:**
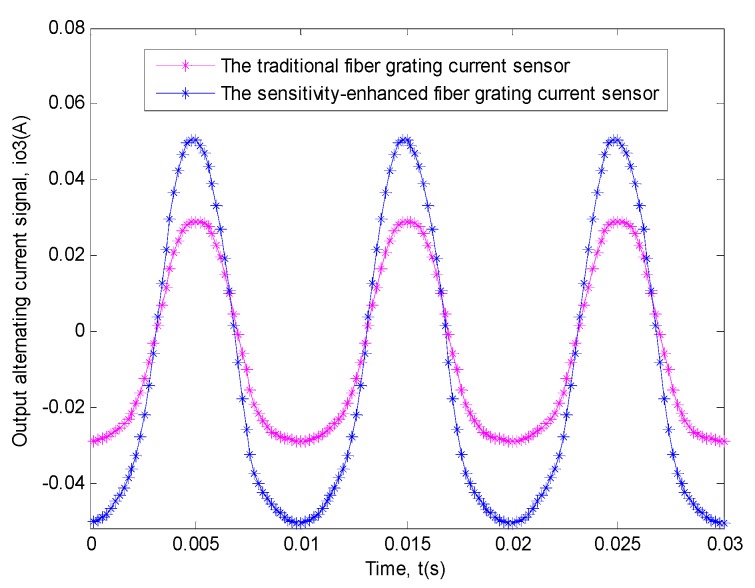
This experiment result of the two sensors between output alternating current signal (*i_o_3__*) and time (*t*). The blue curve shows the relationship of the sensitivity-enhanced fiber grating current sensor. The magenta curve shows the relationship of the traditional fiber grating current sensor.

For the measurement of the power frequency sinusoidal alternating current signal with the amplitude of 262 A, we had done ten experiments and calculated the average of the data from the ten experiments as well. The relationship based on the actual average data obtained by the two sensors between output alternating current signal and time is shown in Figure 12.

**Figure 13:**
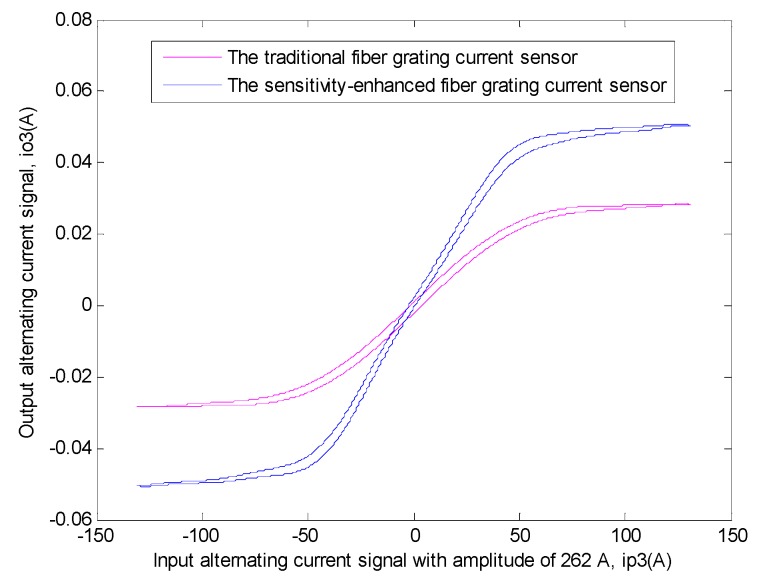
The transfer function relationship curves of the two sensors between output alternating current signal (*i_o_3__*) and input alternating current signal with amplitude of 262 A (*i_p_3__*) respectively. The blue curve shows the transfer function relationship of the sensitivity-enhanced fiber grating current sensor. The magenta curve shows the transfer function relationship of the traditional fiber grating current sensor.

Similarly, in order to explain this problem better, the transfer function relationship curves of the two sensors between the input signal and the output signal need to be drawn [11,12,32,33,34,35,36,37,38], undoubtedly, the method was the same as that used in the first experiment. The specific situation is shown in Figure 13.

Then, the data corresponding to different periodic linear regions in the experimental result of two sensors were also extracted and their average values were calculated [10,11,12,13,28]. The linear region relationship equations of the two sensors between input current signal and output current signal can be obtained by linear fitting with this related software. Their equations are as follows.

This sensitivity-enhanced fiber grating current sensor:

(6) $$\varphi_{1}\left(x_{1}\right)=0.0008311x_{1}$$

Here, when these data are linearly fitted, the value of *R*^2^ is 0.9971, which indicates the best level of fitting at this moment.

This traditional fiber grating current sensor:

(7) $$\varphi_{2}\left(x_{2}\right)=0.0004844x_{2}$$

Here, when these data are linearly fitted, the value of *R*^2^ is 0.9991, which indicates the best level of fitting at this moment.

It can be seen from the above two equations that the linear region slope of the transfer function of the sensitivity-enhanced fiber grating current sensor is 0.0008311, and at the same time, the linear region slope of the transfer function of the traditional fiber grating current sensor is 0.0004844. Then the sensitivity of the sensitivity-enhanced fiber grating current sensor is 71.57% higher than that of the traditional fiber grating current sensor.

**Figure 14:**
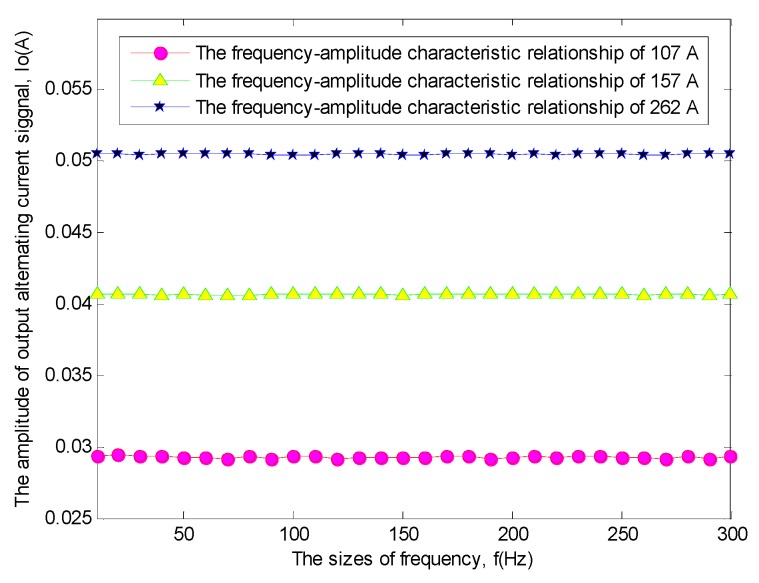
The frequency-amplitude characteristic relationship of this sensitivity-enhanced current sensor. The red curve with some magenta solid circles shows the frequency-amplitude characteristic relationship of the alternating current excitation signal of 107 A. The green curve with some yellow solid triangles shows the frequency-amplitude characteristic relationship of the alternating current excitation signal of 157 A. The blue curve with some black solid five-pointed stars shows the frequency-amplitude characteristic relationship of the alternating current excitation signal of 262 A.

Besides, the frequency-amplitude characteristic is also an important feature of this sensitivity-enhanced current sensor [11,12,13]. Therefore, for the above three current excitation signals, a frequency-amplitude characteristic experiment was implemented, and the frequency-amplitude characteristic relationship picture of this sensitivity-enhanced fiber grating current sensor as shown in Figure 14 is obtained.

It can be seen from Figure 14 that the amplitude of the output alternating current signals doesn’t basically change with the sizes of frequency.

## 4. Explanation and Discussion

(1) The reason that different multiples of sensitivity improvement appear in experimental results when this sensitivity-enhanced fiber grating current sensor is applied to measure different current signals.

**Figure 15:**
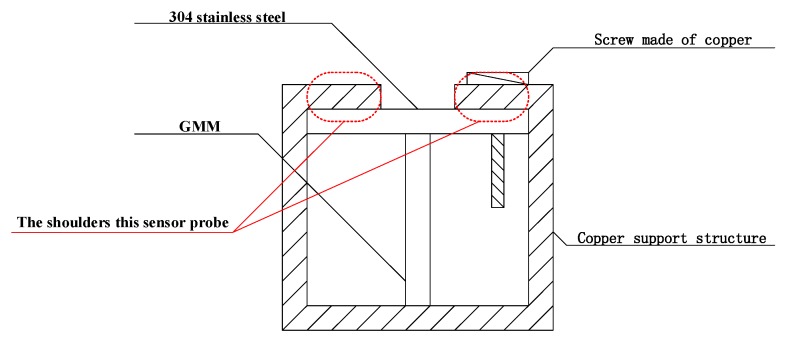
The shoulders of the external support structure of the sensor probe. The area enclosed by two red ovals is the shoulders of this sensor probe.

Through experimental and empirical analysis, it can be concluded that the reason for this phenomenon may be caused by the blocking effect of the shoulders of the external support structure of the sensor probe on the T-shape structure. In fact, the sensitivity of the sensitivity-enhanced fiber grating current sensor can also be understood as the response degree of the sensitivity-enhanced fiber grating current sensor to the weak current signal. However, the response degree also needs to be reflected by the motion degree of GMM, therefore, the external support structure of this sensor probe begins to hinder the movement of GMM to some extent, then the sensitivity of the sensitivity-enhanced fiber grating current sensor is not necessarily increased as expected. The shoulders of the external support structure of the sensor probe of this sensitivity-enhanced fiber grating current sensor are shown in Figure 15.

In addition, the reason for this phenomenon can be simply explained by the traditional cognitive experience. 157A compared to 107A increases the amplitude of the measured current by 50A, at this time, the sensitivity of the sensitivity-enhanced fiber grating current sensor, compared with that of the traditional fiber grating current sensor, is reduced by 133.24%, and 262A compared with 157A increases the amplitude of the measured current by 105A. However, at this time, the sensitivity of the sensor, compared with that of the traditional fiber grating current sensor, is reduced by 64.15%. If it is proportionally changed, it should be reduced by about 260%, but here it is directly reduced by 64.15%, which shows that the hindrance of the shoulder of the external support structure is already great when the amplitude of the measured alternating current is 157A, then the hindered function of the shoulder of the external support structure cannot increase too much when the amplitude of the measured alternating current is 262A as well. and which matches reality. However, all the above mentioned is only based on the guess of empirical theory. Under the limitation of this kind of sensor itself, there is no way to prove this set of empirical theory, because the way to improve the sensitivity of this type of sensor is to design a T-shape structure that is fixed by an external support structure, and GMM is pressed by a special screw on the shoulder of the external support structure, therefore, there is no way to eliminate the obstruction caused by the shoulders of the external support structure, so this set of empirical theories cannot be confirmed. However, this article describes a new type of sensor, and the sensor achieves its purpose of being designed to greatly improve sensitivity, therefore, this sensitivity-enhanced fiber grating current sensor is further optimized after the later stage, then this sensitivity-enhanced fiber grating current sensor must play a big role in this field.

(2) The reason that the percentages are used instead of specifying how much the sensitivities have been increased.

The increased sensitivities are expressed as the percentages rather than a specific increase to values, which indicates that the sensitivities of the sensitivity-enhanced fiber grating current sensor are different when the magnitudes of the measured current are different. Therefore, this brings trouble to the application of the sensitivity-enhanced fiber grating current sensor. If the method of improving the sensitivity of the sensitivity-enhanced fiber grating current sensor is used together with other relatively mature methods of sensitivity enhancement, it is more practical to use the percentages to indicate how much the sensitivities are improved.

(3) The reason that the correspondence between the measured current signals and the output current signals, whose values are so different in size, is not established.

The shoulders of the external support structure of the sensor probe can hinder the movement of the T-shaped structure, therefore, the movement of GMM can be less than expected, which results in the movement of the fiber grating is not as expected (more specific reasons can refer to the above(1)). This ultimately leads to the irregular output current signals for the different measured current signals. Therefore, the reason finally gives rise to an irregular relationship between the measured current signals and the output current signals. In addition, this paper mainly explains the method of sensitivity enhancement, and proves that this sensitivity-enhance fiber grating current sensor can enhance the sensitivity greatly. Of course, it is a focus to establish the correspondence between the measured current signals and the output current signals, which can help users really understand the actual size of the measured current signal, in the future research as well.

(4) The reason that a pressure value is only calibrated to justify the experimental results and only three current values are measured multiple times.

The core of this sensitivity-enhanced fiber grating current sensor is to apply different sizes of prestressing force to GMM, and the experiment is ultimately compared to the case where no prestressing force is applied in GMM, namely, there is only the difference between “prestressing force” and “no prestressing force”, as for how much these prestressing forces are applied, there is no need to overemphasize. Firstly, this is because this is just a proof experiment, in order to prove that this method is successful for improving the sensitivity of this sensitivity-enhanced fiber grating current sensor. Secondly, the jump characteristics of GMM itself make the sensitivities of GMM under different sizes of greatly different prestressing force, namely, when the large size of prestressing force is applied to GMM, the multiple of the sensitivity improvement of GMM is small, which gives rise to the small increased multiple of the sensitivity of the sensitivity-enhanced fiber grating current sensor. Therefore, a prestressing force value can prove that the sensitivity-enhanced fiber grating current sensor can greatly improve the sensitivity, then there is no need to apply many sizes of prestressing force to GMM. After that, we will optimize many aspects of this sensitivity-enhanced fiber grating current sensor, and it is best to find the best this value of prestressing force for this type of sensor, which will make the sensitivity of this sensitivity-enhanced fiber grating current sensor achieve maximum value and this will also be a focus of future research.

The reason that this experiment only carried out multiple experiments on three current values has been clearly stated in the paper. It can be known from the previous description that the changes of the sensitivity-enhanced multiples of the sensitivity-enhanced fiber grating current sensor are not linear for the measurement of different values of the measured current signal, therefore, this makes no sense to measure too much current values. However, this step is still to be carried out in subsequent studies in order to find the optimum range for the current measurement of the sensitivity-enhanced fiber grating current sensor.

## 5. Conclusions

In conclusion, the sensitivity of the sensitivity-enhanced fiber grating current sensor compared with the sensitivity of the traditional fiber grating current sensor is significantly improved. The two sensors, at the same time, were used to measure the power frequency sinusoidal alternating current signals with the amplitude of 107, 157 and 262 A respectively. The sensitivities of the sensitivity-enhanced fiber grating current sensor were 268.96%, 135.72% and 71.57% higher than that of the traditional fiber grating current sensor respectively. In addition, the amplitude of the output alternating current signals of this sensitivity-enhanced fiber grating current sensor doesn’t basically change with the sizes of frequency as well. Moreover, the overall structure and the operation process are simple and efficient, what’s more, the cost is also extremely low. Therefore, this sensitivity-enhanced fiber grating current sensor can simply, practically and cheaply solve the difficult issue which has been plaguing the power industry of high sensitivity and uninterrupted-online measurement for large currents.

## References

[1] Zhao H., Sun F.F., Yang Y.Q., Cao G.Y., Sun K. (2013). A novel temperature-compensated method for FBG-GMM current sensor. Opt. Commun..

[2] Jones C.M., Dai B., Price J., Lin J., Pearl M., Soltmann B., Michael L. (2019). A new multivariate optical computing microelement and miniature sensor for spectroscopic chemical sensing in Harsh Environments: Design, Fabrication, and testing. Sensors.

[3] Du B., Xu X.Z., He J., Guo K.K., Huang W., Zhang F.C., Zhang M., Wang Y.P. (2019). In-Fiber collimator-based Fabry-Perot interferometer with enhanced vibration sensitivity. Sensors.

[4] Li K., Chan T.H.T., Yau M.H., Nguyen T., Thambiratnam D.P. (2013). Very sensitive fiber Bragg grating accelerometer using transverse forces with an easy over-range protection and low cross axial sensitivity. Appl. Opt..

[5] De Nazare F.V.B., Wemeck M.M. (2015). Compact optomagnetic bragg-grating-based current sensor for transmission lines. IEEE Sens. J..

[6] González-Vila A., Ioannou A., Loyez M., Debliquy M., Lahem D., Caucheteur C. (2018). Surface plasmon reasonance sensing in gaseous media with optical fiber grating. Opt. Lett..

[7] Zhang Q., Zhu Y.P., Luo X.Y., Liu G.G., Han M. (2017). Acoustic emission sensor system using a chirped fiber-Bragg-grating Fabry-Perot interferometer and smart feedback control. Opt. Lett..

[8] Yamagata Y., Oshi T., Katsukawa H., Kato S., Sakural Y., Kirkham H., Johnston A.R. (1993). Development of optical current transformers and application to fault location systems for substation. IEEE Trans. Power Deliv..

[9] Zhang L. (2009). Current sensor based on giant magnetostrictive material and fiber Bragg grating. Master’s Thesis.

[10] Yao X.F. (2007). Optical fiber Fabry-Perot current sensor based on giant magnetostrictive materials. Master’s Thesis.

[11] Sun F.F. (2017). Research of GMM fiber current sensor. Ph.D. Thesis.

[12] Liu J. (2013). Study of GMM-FBG current transformer based on DFB laser demodulation technology. Ph.D. Thesis.

[13] Wang L. (2012). Theoretical and experimental studies of current sensing based on giant magnetostrictive materials and fiber grating. Ph.D. Thesis.

[14] Olabi A.G., Grunwald A. (2008). Design and application of magnetostictive materials. Mater. Des..

[15] Clark A.E., Teter J.P., McMasters O.D. (1988). Magnetostriction “jumps” in twinned Tb_0.3_Dy_0.7_Fe_1.9_. J. Appl. Phys..

[16] Clark A.E., Savage H.T., Spano M.L. (1984). Effect of stress on the magnetostriction and magnetization of Single cryatalTb_0.27_Dy_0.73_Fe_2_. IEEE Trans. Magn..

[17] Kendall D., Piercy A.R. (1994). Comparison of the dymic magnetomechanical properties of Tb_0.27_Dy_0.73_Fe_2_ and Tb_0.30_Dy_0.70_Fe_2_. J. Appl. Phys..

[18] Hathaway K.B., Clark A.E., Teter J.P. (1995). Magnetomechanical damping in giant magnetostriction alloys. Metall. Mater. Trans. A.

[19] Wang Z.B., Liu J.H., Jiang C.B., Xu H.B. (2011). The stress dependence of magnetostriction hysteresis in TbDyFe[110] oriented crystal. J. Appl. Phys..

[20] Wang B.W., Busbridge S.C., Guo Z.J., Zhang Z.D. (2003). Magnetization processes and magnetostriction of Tb_0.27_Dy_0.73_Fe_2_ single crystal <110> direction. J. Appl. Phys..

[21] Zhao P., Zhou Y.B. (2012). Magnetoelastic properties dependence on compressive stress in <110> oriented TbDyFe polycrystalline alloys with high drive levels. Mod. Phys. Lett. B.

[22] Wan Y.P., Fang D.N., Hwang K.C. (2003). Non-linear constitutive relations for magnetostrictive materials. Int. J. Non-Linear Mech..

[23] Mei W., Okane T., Umeda T. (1998). Magnetostriction of Tb-Dy-Fe crystals. J. Appl. Phys..

[24] Zhao P. (2009). Experimental investigations of magneto-mechanical coupled characteristics in giant magnetostrictive materials. Ph.D. Thesis.

[25] Moffett M.B., Clark A.E., WunFogle M., Linberg J., Teter J.P., Mclaughlin E.A. (1991). Characterization of Terfenol-D for magnetostrictive transducers. J. Acoust. Soc. Am..

[26] Satpathi D., Moore J.A., Ennis M.G. (2005). Design of a Terfenol-D based fiber-optic current transducer. IEEE Sens. J..

[27] Liang Y.R., Zheng X.J. (2007). Experimental researches on magneto-thermo-mechanical characterize action of Terfenol-D. Acta Mech. Solida Sin..

[28] Xiong Y.L. (2007). Theoretical and technical research on A Terfenol-D and optical Bragg grating based current senor. Ph.D. Thesis.

[29] Rodrigez-Martinez J.A., Pesci R., Rusinek A. (2011). Experimental study on the martensitic transformation in AISI 304 steel sheets subjected to tension under wide ranges of strain rate at room temperature. J. Mater. Sci. Eng. A.

[30] Miu K.N., Chiang H.D. (2000). Electric distribution system load capability: Problem formulation, solution alagorithm, and numerical results. IEEE Trans. Power Deliv..

[31] Chen C.X., Xiang T.Y., Tu G.Y., Tan S.T. (2012). Electrical Engineering Foundation.

[32] Davis M.A., Kersey A.D. (1994). All-fiber Bragg strain-sensor demodulation technique using a wavelength division coupler. Electron. Lett..

[33] Huang Y.H., Lu C., Wai P.K.A., Tam H.Y. (2009). Fast FBG sensor interrogation system using vertical cavity surface emitting laser source. Sens. Actuators.

[34] Kersey A.D., Berkoff T.A., Morey W.W. (1993). Multip-lexedd fiber Bragg grating strain-sensor system with a fiber Fabry-Perot wavelength fiber. Opt. Lett..

[35] Liu K., Liu T.G., Peng G.D., Jiang J.F., Zhang H.X., Jia D.G., Wang Y., Jing W.C., Zhang Y.M. (2010). Theoretical investigation of an optical fiber amplifier loop for intra-cavity and ring-down cavity gas sensing. Sens. Actuators B.

[36] Smith R.C., Dapino M.J., Seelecke S. (2003). Free energy model for hysteresis in magnetostrictive transducers. J. Appl. Phys..

[37] Jiles D.C., Atherton D.L. (1983). Ferromagnetic hysteresis. IEEE Trans. Magn..

[38] Jiles D.C. (1995). Theory of the magnetomechanical effect. J. Phys. D.

